# Merger and Acquisitions Integration, Implementation as Innovative Approach Toward Sustainable Competitive Advantage: A Case Analysis From Chinese Sports Brands

**DOI:** 10.3389/fpsyg.2022.869836

**Published:** 2022-04-26

**Authors:** Wen-Hong Chiu, Yuan-Shen Shih, Li-Sheng Chu, Shieh-Liang Chen

**Affiliations:** ^1^School of Political Science and Law, Jiaying University, Meizhou, China; ^2^Department of Business Administration, Asia University, Taichung, Taiwan

**Keywords:** cross-border mergers and acquisitions, resource-based theory, brand strategy, innovation, competitive advantage

## Abstract

Brand M&A has long been an extremely common strategy for expanding the scale of an organization and entering new business areas, but various signs show that many brand mergers and acquisitions (M&As) do not add value. They often lose money and fail. This research explores the value, scarcity, and non-replicability of resources in corporate M&A, as well as organizational resource management, innovation resource management, and implementation of the combination of resource utilization and brand strategy that incorporate M&As. Taking 03 of China’s sporting shoe industry cases, this study uses the literature to collect, analyze, and organize the conversations of high-level managers to compare and integrate the motivations of corporate M&As to conduct confirmatory analysis. Using case studies and cross-border M&A related secondary data from 2014 to 2021 and supplemented by senior executives’ conversations, 1,836 articles were collected as analysis units. The research results show that Chinese companies’ cross-border M&A’s main corporate strategic motives have four key elements: accelerated expansion, integration of resources, brand integration, rapid entry into the international market, and obstacles to the construction of new entrants. The research results also show that integrating resources and brand execution strategies after M&As correlates to their success or failure. The purpose of the research was first to discuss brand M&As and corporate strategies in the Taiwanese context. Secondly, it discusses the issue of the use of resources by the acquired party in specific to emerging trends in consumer resistance to innovation and acceptance of technological innovativeness in the sports industry brands. Third, it analyzes the effectiveness of brand strategy integration and implementation. Finally, it provides a strategic reference for brand M&As in the industry.

## Introduction

The wave of international mergers and acquisitions (M&As) between companies started in the United States 100 years ago, but up to the present, most of them are not as successful as expected. A large number of academic research cases also show that many M&As fail to realize their forecasted benefits. According to Charles and Gareth (1996), the probability of successful M&As is only about 20%. The main reason for the failure of M&As is that the acquisition cost before the merger is too high, and the internal integration after the merger is not easy, such as culture integration (Barney, 1995). However, under different motives, such as striving to expand the market, obtain unique resources, or reduce various management costs, companies are still making progress worldwide (Wu, 2006). This research advanced the existing literature by exploring the pre-merger considerations and post-merger implementation plans from a resource-based perspective. In addition, to shed light on financial issues, this study helps better understand the motivation for mergers and post-merger integration and execution strategies that affect the success or failure of M&As (Iwao, 2020).

In terms of gross domestic product, Chinese growth was the world’s second-biggest in 2010. Chinese firms are now exploiting their comparative advantage as global players. Developing greater commodities and subsequently enhancing their performance was one of the pillars of that approach (Shih et al., 2019).

**FIGURE 1:**
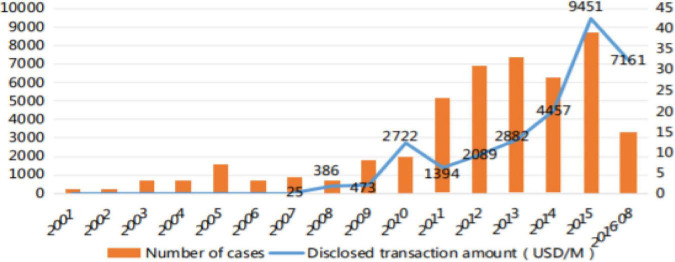
Source (He, 2018; Zhang, 2020).

The important factor was Taiwan’s financial firms’ ability to attract external stakeholders. Products, chemicals, and equipment are Taiwan’s principal exports. In 2001, the number of international M&As of Chinese equipment-manufacturing companies increased, and they may be split into three periods. The first phase starts from “2001 to 2007,” the second phase starts from “2008 to 2013,” and the last phase starts from “2014 to onward,” as explained in Figure 1 below (He, 2018; Zhang, 2020).

The incremental advance of this study was to explore the constructs of value, scarcity, and non-replicability of resources in corporate M&A. As, well as organizational resource management, innovation, and implementation of resource utilization and brand strategy combination incorporate M&As. This research used the resource-based theory to support the associations proposed and tested. The resource-based theory assumes that companies have different tangible and intangible resources transforming into unique resource capabilities (Angwin and Meadows, 2015). This context of Chinese sports brands’ M&A cases was also a unique combination with advancement in theory and evidence related to the explained phenomenon under investigation in this research. Thus besides theoretical advance rooted in the resource-based view mentioned above, this research is incremental for its contextual advance to provide evidence of international mergers and acquisitions from China and Taiwan. Another major advance by this research is to explore the motives of companies’ cross-border M&As and post-merger integration strategies, to enable companies to produce substantial synergies after cross-border M&As. Recently, studies related to consumer resistance to innovation and consumer acceptance of innovation have been debated by several scholarly outlets. Such studies include blockchain technology and sustainability (Friedman and Ormiston, 2022), consumer resistance to innovation in small clothing brands (Ju and Lee, 2020), mobile payment and innovation resistance (Migliore et al., 2022), AI-powered autonomous vehicles, and innovativeness (Meyer-Waarden and Cloarec, 2022). In this scenario, this study on sports brands’ strategy and M&As success factors may bring insightful suggestions for policymakers and scholars in consumer innovation resistance from post-merger and acquisitions perspective.

Therefore, the purpose of this research is as follows. First, it discusses brand M&As and corporate strategies (Liu et al., 2018). Second, it analyzes the issue of using the acquired party’s resources. Third, it investigates the effectiveness of brand strategy integration and implementation. Finally, it provides a strategic reference for an industry’s overseas M&As (Zhang, 2020).

The current study aims to shed light to explore and achieve the following research objectives:

1. Does multiple resource commitment influence the resource-based model?
2. Does the resource base model influence a firm’s implementation and exploitation before and after Merger and acquisition?
3. In the Chinese sports industry, understanding the value, scarcity, and non-replicability of resources incorporated M&A.
4. How corporate strategy and brand strategy implementation and integration is important to determine the success or failure of M&A in a specific context.

## Literature Review

### Cross-Border Mergers and Acquisitions

In a narrow sense, M&As are limited to the transfer of control and cooperative behavior of enterprises that use the acquisition of shares as a means. In a broad sense, M&As refers to everything that involves transferring corporate control and cooperation (Fang-Ming et al., 2006). Acquisitions refer to the purchase of stocks, assets, or business departments from the seller’s enterprise, but not necessarily the purchase of all the seller’s assets and shares held. Past research has shown that acquisitions effectively use existing knowledge and explore new possibilities (Zhang et al., 2020). Acquisitions can be divided into asset acquisitions, equity acquisitions, and mergers according to different acquisition targets. First, the purchase of assets denotes a general asset purchase and sale. The asset purchaser does not have to bear the debts of the acquired company. Second, there is the purchase of stock–that is, purchasing of all or part of the equity of the selected company and incorporating it into the scope of reinvestment business. The selected company continues to operate as an independent legal entity, and the acquirer becomes a shareholder of the selected company. Generally speaking, the acquisition is established after the former acquires 30% of the equity (Zhu et al., 2002). Finally, a merger refers to the combination of two or more companies into one legal entity following the legal procedures of the home country to obtain control of the company by acquiring equity or assets (Wei-Feng et al., 2012).

A merger can be divided into two types: statutory merger and creation merger. A statutory merger, also known as a survival merger, refers to the combination of two or more companies, of which one survives, and the other does not. The surviving company generally bears all the assets and liabilities of the “lost” company. Creation merger, also known as new establishment merger or establishment merger, refers to the combination of two or more companies, in which all companies are dissolved and lost, a new company is established. Then the assets and liabilities of all the original companies are assumed by one entity (Wei-Feng et al., 2012). Dandapani et al. (2020), present results that the shareholders of US companies speak highly of a company’s initial efforts to achieve globalization through international acquisitions. Solberg and Helland (2011), believe that M&As are a strategic tool for companies to pursue rapid growth or diversified operations and one of the strategic practices for companies to continue to have a competitive advantage.

The motives of enterprises using M&As as their external growth path can generally be summarized as follows (Zhang et al., 2020). First, they diversify risks. Through M&As, enterprises expand their business scope and diversify their operations, spreading risks, and stabilizing company operations. Second, they help to pursue growth and increase market share. M&As are a shortcut to growth, which can save the initial losses of a business, quickly obtain the original brand market, and increase market share in a short period. Third, firms seek economies of scale through M&As, enjoy reduced personnel and management expenses brought about by economies of scale, and see enhanced competitiveness. Fourth, there is improved management performance, as mergers among companies with good operating conditions help absorb management methods and business concepts, improve performance, and create a surplus. Fifth, if surplus companies can merge with loss-making companies, they can achieve tax-saving benefits, while loss-making companies can improve their financial status and business strength. Sixth, M&A can increase a company’s market share and capital, relatively improve the ability to bear risks, and at the same time establish the company’s reputation and good image. Seventh, cross-border M&As can help firms obtain technical capabilities that were not available before, use technology and brand assets, and execute new business models and innovative skills. Finally, Salter and Weinhold (1980) note that through M&As, firms can obtain complimentary resources or expand their product lines, and when the value after the merger is higher than the value before the merger, the strategy helps the firms easily achieve their goals. To sum up, this research believes that the key factors motivating M&A are: (1) to accelerate the expansion of the economic scale of enterprises; (2) to save funding into technology research and development; (3) the combination of brands can expand the target audience; and (4) the continuous expansion of scale and construction of new entry barriers.

Wernerfelt (1984) shows that M&As can be described as an incomplete market with low information transparency and a small number of buyers and sellers. Buying resources in batches is one way to maximize returns if a firm buys desired or rare resources. Get the largest potential reward, and go beyond the original area of two or more business M&As, called overseas mergers or cross-border mergers (Christofi et al., 2019; Maung et al., 2019; Dandapani et al., 2020; Paudyal et al., 2021).

### Overseas Merger and Acquisitions in the Sports Industry

Wanda group was the first Chinese group to enter overseas M&As in the sports industry in 2015 (Wang et al., 2021). According to some statistics, about 65% of sporting goods are made in China, and its sporting goods exports ranked 5th in the global ranking (Bai and Shao, 2021). Another international M&As case of collaboration can be reported between FILA and ANTA brands as sporting industry examples of overseas partnerships (Geng and Fan, 2021; Wang et al., 2021). Despite long debate about its effectiveness and desire to adopt M&As strategy, there are only a few cases of success in this industry (Medvid and Zhijiang, 2020). The top 10 brands in the sports industry accounted for 69.1% of the total industry, and none of the Chinese enterprises is listed among those 10 brands. In contrast, the majority belongs to US brands (Medvid and Zhijiang, 2020).

### Pre-post Merger Considerations for Firms

The key factors should be the management’s ability to obtain information and handle interpersonal relationships in the integration phase. Xia et al. (2021) point out that there are three factors for the success of M&A: (1) confirm the target, review, and screen before M&A; (2) a bidding strategy is the most important thing because an appropriate timing of the bid can reduce the price paid to the M&A target; (3) effectively integrate the existing resources of buyers and sellers to achieve the effect of 1 + 1 is greater than 2. Drucker (1981) believes that the five elements that affect the success of corporate M&As are as follows. First, the buyer and seller are technically complementary, creating bilateral benefits. Second, there is a certain degree of relevance or potential in the buyer’s and sellers’ products, markets, and customers. Third, buyers and sellers must have similar corporate cultures to facilitate integration. Fourth, the buyer needs to have senior management personnel who can replace the seller’s enterprise after the merger, in order to control the seller’s resources. Finally, after the merger, the mid-level management of both buyers and sellers must have substantial promotion benefits, which can create a win-win effect.

Burgman (1996) believes that successful M&As are as follows. First, the better the buyer is familiar with the nature of the seller’s business, the greater is the chance of success after cross-border M&As. Second, if buyer management can stay in office, then cross-border M&As will have a fairly high chance of success. Finally, the larger the economic scale of the transaction is, the greater is the chance of successful cross-border M&As. The buyer should be cautious about the following matters before a merger. First, what assets does the target company own? Second, which of the target companies merged can effectively gain advantages? Third, what costs should be paid attention to in M&As? Finally, what price does the buyer need to pay for this merger?

Therefore, this study believes that the cross-border M&A process can be divided into four stages: planning stage, negotiation stage, execution stage, and integration stage. In summary, cross-border M&As are defined as all activities involving the transfer of control and command power and the coordination and cooperation of enterprises. This study believes that the most important factors for the success of cross-border M&As are the integration of the culture of the buyer and the seller and the retention and execution capabilities of the seller’s middle- and high-level managers.

### Resource-Based Theory

From the resource-based theory perspective, resources, and products are two sides of an enterprise. Most products require integrating several resources to complete, and a single resource can also be used for several products. The resource-based theory assumes that companies have different tangible and intangible resources that transform into unique capabilities (Wernerfelt, 1984; Angwin and Meadows, 2015). Therefore, these unique resource capabilities are the source of strengthening the enterprise’s competitive advantage. These resources generate high profits and high remuneration potential from superior production experience, technological leadership, customer loyalty, and M&A (Wernerfelt, 1984). The achievement of a corporate strategy depends on the development and utilization of resources.

**FIGURE 2:**
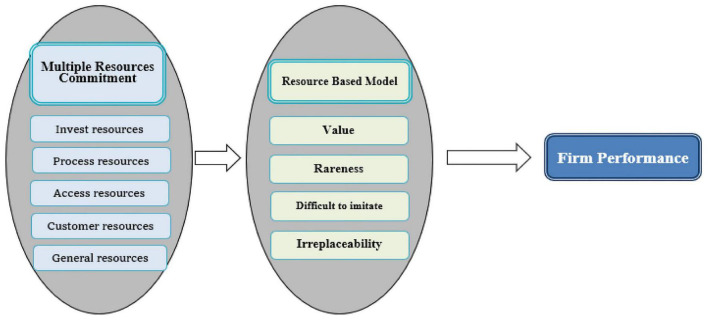
Analysis framework based on resource-based theory.

As highlighted in Figure 2 above, a company requires asset operations, customer relationships, creation and provision of value, and revenue, including the cost of acquiring key resources, partnerships, and activities (Meurs et al., 2020). Verdin and Williamson (1992) divide resources into five categories: (1) input resources: raw materials, production capacity, the relationships between equipment and suppliers, etc.; (2) process resources: R&D capabilities, technical knowledge management, employee process experience, etc.; (3) channel resources: distribution network, agent loyalty, market share, etc.; (4) customer resources: customer loyalty, brand awareness, after-sales service network, etc.; and (5) ordinary resources: human resource management skills, company, and special industry experience, managerial knowledge, market knowledge analysis ability, financial resources, information system integration ability, and a relationship with the government (Zollo and Meier, 2008; Bettinazzi and Zollo, 2017). In summary, the study finds that organizational resources in the resource-based theory should have four characteristics before having a competitive advantage: (1) Scarcity, (2) Uniqueness, (3) Cannot be imitated, and (4) Cannot be replaced. Therefore, the effectiveness of organizational resources lies in how the company counts resources, effectively utilizes resources and allocates them appropriately to maximize profits. Therefore, we believe that corporate resources include all assets, knowledge, capabilities, corporate characteristics, organizational processes, information systems, etc.

### Brand Strategy

Usually, a brand prioritizes quality and has cultural and emotional connotations. Thus, the brand improves the added value of the product. At the same time, the brand has a certain degree of trust and loyalty, and the company can set a relatively high price for the brand with a liposuction strategy and obtain higher profits (See Berry). Well-known brands perform most prominently in this aspect, such as Adidas sneakers, which are several hundred yuan higher than Li Ning and Anta sneakers. A company can decide to use a different brand for each product. Adopting individual brand names and seeking different market positioning for each product is beneficial to increased sales and fighting against competitors. A firm can also diversify risks and separate the success or failure of individual products from the company’s reputation so that the company’s entire reputation will not be affected by poor performance. A brand is a total collection of target consumers’ psychology, comprehensive feelings, and perceptions of a particular thing. In marketing, the main function is to differentiate the company’s products and services from competitors (Meurs et al., 2020). A brand is a name or a term, symbol, mark or pattern, or an optional combination used to identify a product or service for a certain scale or group of consumers.

Brand management can bring business growth and benefits to the company, and a good brand image can also create a long-term competitive advantage in the market (Keller et al., 2008). Examined a luxurious environment and showed that brand aura could produce abnormal behavior. Haenggli and Hirschi (2020) evaluate the relationship between key resources, knowledge/skills, and their predictive utility for different forms of subjective and objective career success (i.e., salary). Zhu et al. (2002) state that sales people with brand recognition capabilities have a high ability to resist changes. On the other hand, salespeople with brand recognition will develop a brand-centric method, but rarely a customer-oriented sales method. Wider et al. (2018) present that stakeholders work with brand management to shape the brand by participating in brand-related interactions. Erkollar and Oberer (2016) Opportunities for brand size and brand environment. Erkollar and Oberer (2016), the correlation test shows a positive correlation between brand management and brand citizenship behavior. Bettinazzi and Zollo (2017) state that consumer attachment closely relates to brand loyalty. Thus consumer resistance and acceptance may have significant implications for brands that are adopting M&As as a strategy, and the current study is an attempt to discuss success and failure factors for adopting such a strategy.

As per the study setting of Taiwan and the relationship between brand strategy specific to M&As in Taiwan, very few studies have addressed it directly or indirectly. It warrants this research as an incremental advance to existing literature. A recent study conducted in a similar context on green M&As in Chinese and Taiwanese contexts has reported the significant influence of such strategies on brand innovations, organizational performance, and cross-border success (Lu, 2022). Another recent research on strategic green marketing and the role of M&As strategy in the Taiwanese context reported that some external factors such as financial advisory may affect the M&As strategy and green marketing approach relationships (Zhu et al., 2002; Pérez-Pérez et al., 2021). Such results encouraged the authors to examine the associations conceptually under investigation in this study.

## Research Methodology

This research adopts a case study method, focusing on three emerging sports brands (Anta, Li Ning, and Xtep) that have laid a foundation for development in China, gradually moved into the international sporting goods industry, and constructed corporate cross-border M&As with brand M&A motivation and sustainable management. Integrate the exploratory main axis, collect the cross-border M&As data of these three companies, discuss and analyze the literature on M&As and sustainable operations, and establish the consideration factors and execution results of M&As. Try to analyze enterprises’ strategic thinking and post-merger integration strategy from the theoretical point of view of resource capacity utilization and value allocation of enterprises in M & M&As. According to Yin (1994), scholars believe that whether for the investigation method, experimental method, archive analysis method, historical research method, or case study method, they can all be used in exploratory, descriptive, and explanatory research. As mentioned in Table 1 below each method has its characteristics. For the advantages and disadvantages and the applicable situation, whatever method is adopted, the researcher should consider the following points: (1) The nature of the research problem; (2) The researcher’s controllability of the research phenomenon; and (3). Finally, research phenomena are contemporaneous or non-contemporaneous things.

**TABLE 1:** Related status of different research method strategies.

Research method	Survey	Experimental Method	File analysis	Historical research method	Case study
Research problem traits	Who?	Who?	Who?	Who?	Who?
	What is it?	What is it?	What is it?	What is it?	What is it?
	How many?		How many?		
	Where?		Where?		
Research variables	No	Yes	No	No	No
Degree of control					
Whether it is at the same time period issues	Yes	Yes	Yes/No	No	No

## Case Analysis and Discussion

This study takes Chinese sports brand companies as the research case objects. The companies are Anta, Xtep, and Li Ning. In April 2017, China’s local sports leading brands announced their 2016 financial reports. Among them, Anta’s total revenue was RMB 13.35 billion, increasing 20% year on year, breaking the ten billion revenue mark for Chinese sports brands. Li Ning increased by 13.1%, and Xtep increased by 1.9%. Anta 48.4%, Li Ning 46.2%, and Xtep 43.2% in gross profit. A summary of the case for cross-border M&As, resource utilization, and brand strategy follows below.

### China Anta Group

Anta, in 2009, realized that a single brand structure is difficult to cope with market shocks, so it acquired the franchise and trademark use rights of FILA, one of the world’s top ten sports brands, for HK$600 million. It became responsible for using the trademark in Mainland China, Hong Kong, and Macau and promoting and distributing FILA products. In 2017, it acquired Amer Sports for US$5.2 billion and 58% of its equity. Due to the continuous acquisition of international brands, Anta Group expanded rapidly and entered the international market. In October 2014, Anta became an official NBA market partner and NBA licensee. In 2015, it acquired Sprandi, a British outdoor brand. In 2017, it acquired the brand Kolon Sport from South Korea. In 2017, it acquired Kingkow, a mid-to-high-end children’s clothing brand in Hong Kong. According to a UBS research report, Anta will complete two acquisitions a year through FILA in the next 3 years. All FILA in China belong to Anta Group. Although many people look down upon this sports brand, it is called “Chinese NIKE” in the industry. Anta Group’s market value went as high as HK$97.7 billion, or far ahead of Xtep, Jordan, and other brands (2017-11-10 by Kandian facts published in Finance). According to Anta’s financial report for the first half of 2017, FILA’s revenue accounted for 20% of Anta Group’s revenue. Anta Group has rapidly expanded and established barriers for new entrants by continuously acquiring international brands and implementing an omnichannel strategy.

To further strengthen brand differentiation and industry leadership and lay a solid foundation for long-term brand strategy, in 2013, it announced that the company would once again cooperate with the Chinese Olympic Committee from 2013 to 2016, making Anta become the company for eight consecutive years since 2009. “Sports Apparel Partner of the Chinese Olympic Committee.” The successful renewal of the contract with the Chinese Olympic Committee and the maintenance of a good cooperative relationship fully prove that the Chinese Olympic Committee has full trust in the company’s comprehensive strength and service capabilities. Top Chinese athletes have also recognized its brand image and the quality of its innovative products. Anta Sports has worked closely with the Chinese Olympic Committee to develop a full range of integrated marketing strategies around important events. While effectively promoting the Anta brand, it also vigorously has promoted the development of China’s sports industry and the spread of the Olympic spirit. As for the cooperation period from 2013 to 2016, deeply explore and fully develop high-quality sports resources, formulate a 4-year Olympic plan to promote ANTA Sports and the Chinese Olympic Committee to stimulate growth together, and join hands with the Chinese Olympic Committee to enter the “Sports Age.” During these events, ANTA Sports will also provide Chinese sports delegations with better award-receiving equipment and more professional services to show the world the strength and style of Chinese sports and Chinese sporting goods brands.

Due to the improvement of China’s domestic living standards and the popularization of national sports, consumers’ demand for sporting goods with different functions and designs has been promoted. At the same time, a variety of marketing channels has emerged in response to the rapid changes in the retail market and domestic consumer demand. Anta has always focused on the sporting goods market and adopted a multi-brand and omnichannel strategy to seize opportunities from the Chinese people in the high-end market and important retail channels to promote growth in different market segments. A complete brand portfolio model not only helps Anta prevent market instability but also strengthens its competitive advantage and achieves long-term and sustainable development. The famous British brand evaluation agency Brand Finance released the world’s 50 most valuable apparel brands in 2017. Anta and jewelry brand Chow Tai Fook were the only Chinese brands. Anta is the second-fastest-growing brand on the list, with a brand value of US$2 billion. Especially after the completion of the acquisition of Kingkow, Anta’s share price rose in response, and its current market value is about HK$97.7 billion. It will soon become China’s second apparel group with a market value of over HK100 billion after Shenzhou International. In addition, Anta is also the first sponsored brand of CBA and the first brand to have a national sports laboratory. Anta has grown into a huge apparel giant, leading the domestic sports brands, and it is worthy of having the title of “China Nike.”

### China Xtep Group

As stated in the company’s 2018 annual report, the company’s vision is to expand from a single-brand company to a multi-brand portfolio group. It can be seen that Xtep has a clear willingness to seek M&A opportunities. On April 17, 2019, according to the Korean Herald and other South Korean media reports, the Chinese sports brand Xtep reached an agreement with the Korean apparel retailer group E-Land World to acquire the group’s US footwear brand K-Swiss. It was reported that the capital scale of the acquisition was about 300 billion won or about 1.77 billion yuan. In August 2019, China-based Xtep International Holdings Co., Ltd. announced that it had reached an agreement with E-Land World Company Ltd. and E-Land U.S.A. Holdings Inc. to acquire all of E-Land Footwear U.S.A. Holdings Inc. for US$260 million in issued shares (E-Land Footwear U.S.A. Holdings is the parent company of sports and lifestyle brands K-Swiss, Palladium, and Supra). It took 3 months to complete the three international brands’ acquisition finally. With the United States domestic market as the center, Geshiwei has branch offices in Canada, Germany, the United Kingdom, Singapore, Japan, and other European, American, and Asian countries.

According to Xtep’s official introduction, K-Swiss, a traditional sports shoe brand founded in California in 1966, is committed to providing high-performance tennis shoes and leisure and fitness footwear products to meet the needs of world-class athletes and trendsetters. Palladium, founded in France in 1947, is now one of the world’s most well-known military boot brands. These brands are currently in more than 80 countries and regions worldwide. In March 2019, Xtep announced an agreement with its joint venture partner Wolverine. Both parties agreed to pay an initial capital of 155 million yuan to develop, market, and distribute footwear, clothing, and accessories under Merrell and Saucony brands in Mainland China, Hong Kong, and Macau. The acquisition of E-Land Footwear U.S.A. Holdings Inc. means that Xtep will own all the rights and interests of its brands in the world. With Anta’s previous experience in successfully operating FILA in China and acquiring Finland’s Amer Sports, Xtep seems to want to imitate Anta’s acquisition type, achieving domestic operations of multiple brands and acquiring international brands to take the path of internationalization.

The group is mainly engaged in general sports, high-performance sports, all-terrain adventure activities, and fitness activities, footwear, and clothing for three internationally renowned brands (K-Swiss, Palladium, and Supra) and two sub-brands (PLDM and KR3W) as well as accessories and casual wear design, development, and marketing. Xtep said that the acquisitions are an excellent opportunity to invest in a series of world-renowned sports and leisure brands for the high-end market. These brands have unique brand positioning and target different consumer groups, complementing Xtep’s brand resource portfolio. The acquisition will promote Xtep’s transformation into a global sporting goods company that can meet the needs of different consumers, and these brands can benefit from Xtep’s huge sales network resources, leading R&D technology resources, and supply chain resources, enabling them to operate at high speeds. Full potential in the growing sporting goods market in Greater China.

### China Lining Group

In May 2020, China Sporting Goods Li Ning’s major shareholder, Viva China, controlled by the Li Ning family, announced that it would acquire a 66.6% stake in Bossini for 46.62 million yuan. Viva China’s board of directors believes that despite the downward trend in the retail industry in Hong Kong and Macau, Bossini is a well-known apparel brand in the industry, and Hong Kong and Macau are still world-renowned retail and tourism destinations, which guarantee a large customer base. Bossini Group currently has 180 directly-operated stores in China (mainly in Guangdong Province), and Viva China Group believes that Bossini Group can further promote its brand in China and seize business opportunities in China. Coupled with the management team of the group’s consumer goods business has extensive experience in the Chinese apparel industry; Viva China’s board of directors still maintains a positive attitude toward Bossin’s prospects (especially the development potential in China) and believes that the outlook for the apparel industry is still bright in the long run. In October 2020, Lion Rock Capital, a Hong Kong private equity fund company, began negotiating to acquire shares in Clarks, a century-old British footwear brand. It is worth noting that the current chairman of Lion Rock Capital is Li Ning, the founder of domestic sportswear giant Li Ning. This means that Li Ning may be the initiator of Lion Rock Capital’s acquisition of Clarks and the biggest beneficiary. Before this, Li Ning had made many acquisitions, but compared with Anta’s great success in acquiring FILA’s business in Greater China, the results of Li Ning’s acquisitions were much inferior. The acquisition of brands remained at the scale of Double Happiness and the badminton brand Kai Sheng. Therefore, Lion Rock Capital’s bid to acquire Clarks is considered another move by Li Ning Group to expand its fashion following the acquisition of Hong Kong-owned apparel brand Bao Shilong at the beginning of the year.

China’s huge population also gave birth to new and cutting-edge brands that have sprung up. Better technology and more resources can strongly impact the world. Li Ning is the same, and Li Ning is neither a pioneer nor alone going international. There are many well-known designers such as SANKUANZ in front of Li Ning! The representatives of the same period also include brands such as MYGE and UMAMIISM.

In contrast, these real new-generation brands’ authentic cultural colors have also impacted the world’s vision! In addition to the dazzling clothing items of Li Ning, the ambitious Chinese Li Ning has not disappointed the “sneakers” of his old business and even made many foreign media scornful. Nowadays, trendy Dad Shoes, avant-garde designed running shoes, and excessive levels of stacking Or a unique silhouette, the 30-year-old “sports brand” does not fear the stereotype of the Yangtze River behind the waves and pushes forward and will shine the sun and moon essence absorbed along the way on a show! The bold and avant-garde use of colors is not inferior to the current fashion darlings. I have to praise Li Ning for giving out fascinating items this time. It fits the theme and has new ideas.

Today’s young people can travel the world with their mobile phones. Swipe the page twice and wait for the goods to be picked up at home. If you rewrite the previous “world martial arts, indestructible and unbreakable,” maybe whoever masters the speed in the fashion industry will be half of the success. In 2012, Li Ning suffered a huge loss of two billion RMB, high-end inventory, and many closed stores, putting the firm in trouble. At this time, the founder Li Ning came back, and it took 3 years to turn losses into profits. Starting nationally, the market value has soared by hundreds of billions of RMB in 6 years. Li Ning’s 2020 annual report had revenue of 14.4 billion RMB, gross profit of seven billion RMB, and a gross profit margin of 49%. Li Ning adopts a multi-brand business development strategy.

In addition to its core Li Ning brand (LI-NING), it also owns LOTTO, AIGLE, and Z-DO. In addition, Li Ning has a controlling stake in Shanghai Double Happiness and a wholly-owned acquisition of Kaisheng Sports. Founded in 1989 by Li Ning, the “Prince of Gymnastics” of the same name, his active investment in various sports events has established a deep-rooted sports brand image. Group Executive Chairman Li Ning said: “In 2020, China’s economy will be the first to recover from the new crown pneumonia epidemic. Although the sports industry will be under short-term pressure during the epidemic, with the support and promotion of national policies and governments at all levels, China’s sports consumer market will continue to remain active. In this context, we continue to focus on the value of Li-Ning’s experience, optimize marketing strategies based on the individual needs of consumers, and continue to improve the consumer experience. At the same time, we strengthen the core of our brand and products, focus on building a professional reputation, and continue to interpret trends, keep pace with the times.” Li Ning’s success is mainly due to the company’s breaking out of the dead-end of the previous homogenization of sports brands, completely breaking previous design concepts, and giving Li Ning a trend-seeking Chinese target.

To sum up the above cases, Table 2 lists the corporate brand overseas M&As, the resource base utilization, and the integrated implementation plan of the brand strategies.

**TABLE 2:** The corporate brand overseas M&As.

	China Anta	China Xtep	China lining
Cross-border M&As	1. Rapidly expand the market territory. 2. Implement a multi-brand omnichannel strategy. 3. Obstacles of new entrants threshold.	1. Move toward internationalization. 2. Accelerate the preparation of business development in local and overseas markets	The design brews huge Ambition. Chinese cultural impact on the world perspective.
Resource-based utilization	1. Use credible official resources to become an official NBA market partner and NBA licensee formally. 2. From 2013 to 2016, cooperate with the Chinese Olympic Committee.	1. Purchase when the seller’s loss decreases and the purchase price is reasonable. 2. Xtep’s brand portfolio is highly complementary to a professional leap. 3. Combine sales network resources, R&D technical resources, and supply chain resources.	1. Trendy Dad Shoes, avant-garde designed running shoes, excessive levels of stacking, or unique silhouettes. 2. Show now, Buy to overcome the problem of time difference.
Brand strategy	1. It is difficult for a single brand to cope with market competition and shocks. 2. Continue to acquire international brands. 3. Improve the brand value and market value.	1. One of the most famous military boot brands in the world. 2. Based on the local international strategy. 3. The combination of brand resources is highly complementary.	Multi-brand business development strategy to strengthen brand marketing capabilities.

## Managerial and Theoretical Implications

This section contributes to the research by discussing managerial and theoretical implications drawn from this research and future research avenues that emerged from this study found.

### Theoretical Implications

The new finding has many theoretical ramifications. The resource-based theory first demonstrated that resources and products are two sides of an enterprise. Most products require integrating several resources to complete, and a single resource can also be used for several products. The resource-based theory assumes that companies have different tangible and intangible resources, which can be transformed into unique resource capabilities (Wernerfelt, 1984; Angwin and Meadows, 2015). The effectiveness of organizational resources lies in how the company counts resources, effectively utilize resources and allocates them appropriately to maximize profits. To sum up, this research defines the resource-based theory as having four characteristics that determine the lasting competitive advantage of resources: (1) Value, (2) Rareness, (3) Difficult to imitate, and (4) Irreplaceable. Therefore, we believe that corporate resources include all assets, knowledge, capabilities, corporate characteristics, organizational processes, information systems, etc. (Haenggli and Hirschi, 2020; Ding et al., 2021).

Using a resource-based view to explore the success and failure aspects of M&As is a unique contribution of this research. This has bridged the gap between corporate governance and brand management theories and advanced the research related to brand acquisitions and their effects on competitive advantage and market shares. This study is theoretically incremental to consider resource-based view in sports and apparel brands cases of Chines origin. This contextual importance is a major advance to the field, opening new debates in the global competitive potential of Chinese companies.

Another major theoretical advance by this research is to bridge the gap between M&As is “the purpose of the research is first to discuss brand M&As and corporate strategies,” “use of resources by the acquired party,” and “analyzes the effectiveness of brand strategy integration and implementation.” This research also implies that companies should consider how investors react to risky actions like expansion purchases. While some of the advantages of high-risk trades take time to emerge, traders and investors are sometimes unwilling. Such conditions encourage management scholars to incorporate risk theories with resource-based theories to understand better the risk involved in implementing such strategies.

This research further extended the theoretical landscape of resource-based view in M&As brand strategy to consider consumers’ resistance and adoption of technological innovation while competing in global and local markets.

### Practical Implications

In addition, the new study provides policymakers with useful information in various ways. First, after discussing the motivations of cross-border M&As, individual companies appear to have the following factors: rapid market expansion, implementation of a multi-brand Omni-channel strategy, and construction of barriers for new entrants. Few researchers have suggested the well-studied key aspect in determining the issue of using the resources of the acquired party; before the acquisition, the internal resources of the acquired party should be investigated and researched to integrate total resource use so that the resources of both parties can be properly cross-utilized and maximize resource efficiency (Tsai, 2014).

As a result, administrators and policymakers should look for three criteria while implementing the strategies; in regards to the effectiveness of brand strategy integration and implementation, a strategy implementation after M&As should take into account the strategic positioning of the brand to achieve the brand promotion effect and enhance the loyalty of consumers. As a result, administrators and policymakers should look for three criteria while implementing the strategic reference for an industry’s overseas M&As; the resource base positioning under overseas M&As should be based on strategic positioning to formulate resource competition strategies effectively and obtain a permanent resource competitive advantage (Tsai, 2014; Liu et al., 2018; Meurs et al., 2020; Paudyal et al., 2021).

This research brings several key policy insights for the brand managers and top management of companies who are aggressively looking for M&As as a competitive advantage strategy. Showing enormous growth of Chinese brands based on such implementation strategies brings insights into the global competitive landscape of sports and apparel brands, but these insights can also be extended to other industries and sectors where firms are looking for aggressive M & M&As. This research is vital in terms of cases examples of Chinese brands to show the M&As strategies adopted and successful growth achieved. In broader terms, it also expresses how the Chinese economic model is achieving success in various industries to grab major global shares. This research has opened several new future research avenues in corporate governance and brands management domains.

### Future Directions and Limitations

Similar to all other studies, the current study also includes some drawbacks that must be addressed in future research attempts and its many positives. This research puts forward the following suggestions. First, companies planning to conduct overseas M&As in the sporting goods industry should use their resource advantages to analyze the overseas environment and effectively utilize those same strategies to obtain a competitive advantage. Companies can use their own and external resources to analyze the data in future research. Second, M&As should have the effect of 1 + 1 > 2 to demonstrate their effectiveness and success and only use three companies to conduct data. Future research uses different companies and longitudinal research data for better causality. Thirdly, this research is simple and exploratory to understand the integration and implementation of M&As strategies at the nascent stage. Future studies are recommended to use more robust methodological approaches with mixed methods and other analytical tools to dig deep for key policy insights for the scholars and practitioners in this field. This research only used the case method to compare the Chinese brands, while future scholars may attempt to make comparative case analyses of Chinese and Non-Chinese companies to highlight strategic differences among various companies based on their origin. Similar studies are also recommended in other sectors for better learning outcomes. Future studies may also specifically explore the M&As strategy and consumer resistance and acceptance to technological innovativeness in the specific market before and after mergers and acquisitions of specific brands.

## Conclusion

This study has three conclusions based on the collection, integration, and analysis of case data. First, cross-border M&As make good use of environmental resources and integration processes. The three group companies have utilized their resource-based advantages, catered to the external environment’s opportunities, built a unique corporate culture, and established sustainable competitive advantages. Anta Group aims to expand its territory rapidly and establish an entry barrier for potential competitors with a multi-brand omnichannel strategic model. Xtep Group, with its determination to move toward internationalization, is accelerating business expansion in China and overseas. Li Ning Group focuses on steady design, has huge ambitions, and matches Chinese cultural influence to impact the world’s perspective. All three strategies adopted provide key lessons for students in this field and managers.

Second, in terms of resource base utilization, firms can use a national emotional atmosphere to develop unique competitive advantages of Chinese resources. Anta Group uses the advantages of official credibility to form its beneficial resources to provide consumers with strong brand recognition. Xtep Group acquires a firm when the seller’s losses are reduced, the purchase price is reasonable, and the acquisition cost is reduced by adopting this strategy. Xtep’s brand portfolio has a highly complementary and professional leap. Li Ning Group leads the trend in design and incorporates avant-garde design running shoes with Chinese cultural characteristics. The exaggerated stacking and unique silhouette are the characteristics of Li Ning running shoes, which attract a large number of loyal, young followers.

Finally, in terms of brand strategy: find your niche market through brand positioning. Anta Group understands that it is difficult for a single brand to cope with market competition and shocks, so it continues to acquire international brands to increase brand value and market value. Xtep Group is one of the world’s most well-known military boot brands, based on China’s local international strategy. Li Ning Group strengthened its brand marketing capabilities through a multi-brand business development strategy. Conclusively, this research is incremental to bring several key policy insights for marketers, brand managers, CEOs, and corporate governance scholars.

## Author Contributions

W-HC: supervision and data collection. Y-SS: original writing, contribution of all data, literature improvement, and final version editing. All authors have read and agreed to the published version of the manuscript.

## Conflict of Interest

The authors declare that the research was conducted in the absence of any commercial or financial relationships that could be construed as a potential conflict of interest.

## Publisher’s Note

All claims expressed in this article are solely those of the authors and do not necessarily represent those of their affiliated organizations, or those of the publisher, the editors and the reviewers. Any product that may be evaluated in this article, or claim that may be made by its manufacturer, is not guaranteed or endorsed by the publisher.
